# Transitioning Lessons Learned and Assets of the Global Polio Eradication Initiative to Global and Regional Measles and Rubella Elimination

**DOI:** 10.1093/infdis/jix112

**Published:** 2017-07-01

**Authors:** Katrina Kretsinger, Peter Strebel, Robert Kezaala, James L. Goodson

**Affiliations:** 1 Expanded Program on Immunization, Immunizations, Vaccines, and Biologicals Department, World Health Organization, Geneva, Switzerland;; 2 Health Section, Program Division, United Nations Children’s Fund, New York, New York; and; 3 Global Immunization Division, Center for Global Health, Centers for Disease Control and Prevention, Atlanta, Georgia

**Keywords:** Measles, rubella, poliomyelitis, vaccine-preventable diseases, polio transition, polio legacy.

## Abstract

The Global Polio Eradication Initiative has built an extensive infrastructure with capabilities and resources that should be transitioned to measles and rubella elimination efforts. Measles continues to be a major cause of child mortality globally, and rubella continues to be the leading infectious cause of birth defects. Measles and rubella eradication is feasible and cost saving. The obvious similarities in strategies between polio elimination and measles and rubella elimination include the use of an extensive surveillance and laboratory network, outbreak preparedness and response, extensive communications and social mobilization networks, and the need for periodic supplementary immunization activities. Polio staff and resources are already connected with those of measles and rubella, and transitioning existing capabilities to measles and rubella elimination efforts allows for optimized use of resources and the best opportunity to incorporate important lessons learned from polio eradication, and polio resources are concentrated in the countries with the highest burden of measles and rubella. Measles and rubella elimination strategies rely heavily on achieving and maintaining high vaccination coverage through the routine immunization activity infrastructure, thus creating synergies with immunization systems approaches, in what is termed a “diagonal approach.”

The world is now closer than ever before to achieving global polio eradication. Ridding the world of polio will be our greatest public health success since smallpox eradication and will further cement the critical role of vaccines in achieving public health goals. Since the widespread use of vaccines worldwide began through the global Expanded Program on Immunization (EPI), which started in 1974 following implementation of the Smallpox Eradication Program, vaccines have been widely recognized for their value as the greatest single investment that can be made for improving people’s lives and public health [1, 2]. During nearly 30 years of operations, the Global Polio Eradication Initiative (GPEI) has recruited and trained millions of volunteers, social mobilizers, and health workers; accessed households untouched by other health initiatives; mapped and brought health interventions to chronically neglected and underserved communities; and established a standardized, real-time global surveillance and response capacity [3]. As the initiative nears completion, there is an opportunity and obligation to build a better future by applying the lessons learned from the GPEI and adapting its current infrastructure and unique functions to other global health priorities and initiatives. These priorities should include strengthening the infrastructure for delivery of routine immunization services, control of other vaccine-preventable diseases (VPDs), and measles and rubella elimination (which refers to the reduction to 0 [or a very low defined target rate of] new cases in a defined geographical area, as opposed to eradication, which refers to the complete and permanent worldwide reduction to 0 new cases of the disease through deliberate efforts).

The rationale for pivoting from polio eradication to measles and rubella elimination is compelling. Today, measles continues to be a major cause of child mortality globally [4], rubella continues to be the leading infectious cause of birth defects [5], and measles and rubella elimination goals are established [6, 7]. Measles and rubella eradication (global elimination) is feasible and cost saving [8]. Furthermore, the strategies used by the GPEI and refined through innovations are similar to those needed for measles and rubella elimination, and currently polio resources are concentrated in the countries with the highest measles and rubella burden. Although measles and rubella elimination strategies are similar to those used for polio eradication, the elimination strategies for measles and rubella rely more heavily on vaccine doses administered through routine immunization service delivery. Therefore, embracing measles and rubella elimination would not create another vertical eradication program, but rather would synergistically strengthen immunization systems for all delivering all vaccines. Finally, measles and rubella control and elimination efforts currently depend critically on substantial support from GPEI resources, and a failure to refocus these resources on measles and rubella would cause significant backsliding in already realized gains.

## MEASLES AND RUBELLA ELIMINATION AND IMMUNIZATION SYSTEM STRENGTHENING ARE THE MOST OBVIOUS FRONTLINE CANDIDATES FOR TRANSITIONING OF POLIO ASSETS

The primary goals of polio transition planning are to protect a polio-free world and ensure that investments made to eradicate polio will continue to contribute to future public health goals after the completion of polio eradication. Polio transition planning aims to benefit all countries and the global community, rather than countries in which polio resources are currently concentrated, to achieve common goals. A priority of transition planning is to enable long-term transitions to country ownership of basic public health functions wherever possible. To be effective and sustainable, future transition opportunities for polio program resources should build from and contribute to existing global and regional goals, as well as national or subnational health strategies and goals.

In 2010, the World Health Assembly set 3 targets for measles control by 2015 as milestones toward the global eradication of measles: (1) increase routine coverage with the first dose of measles-containing vaccine (MCV1) for children aged 1 year to ≥90% nationally and ≥80% in every district; (2) reduce the global annual measles incidence to <5 cases per 1 million population; and (3) reduce the global measles mortality by 95% from the 2000 estimate. In 2012, the World Health Assembly approved the key document that serves as the global guide for immunizations in the world today, the Global Vaccine Action Plan (GVAP) [6]. The GVAP set the goal to achieve measles elimination in 4 regions by 2015 and measles and rubella elimination in at least 5 of 6 World Health Organization (WHO) regions by 2020. All 6 WHO regions have established measles elimination goals, and 3 regions have rubella elimination goals. In addition to these measles and rubella goals, Sustainable Development Goal 3.2 aims to end preventable deaths of newborns and children <5 years of age, with all countries aiming to reduce neonatal mortality to ≤12 deaths per 1000 live births and mortality among children aged <5 years to ≤25 deaths per 1000 live births by 2030 [9]. Sustainable Development Goal 3.2 is designed to build on the United Nations Millennium Development Goal 4 (MDG4) to reduce the overall number of deaths among children; routine MCV1 coverage was used as an indicator of progress toward MDG4 [5]. While substantial progress has been made toward these goals since 2000, the World Health Assembly 2015 global control milestones and regional measles elimination goals were not achieved, global MCV1 coverage has not increased substantially in recent years, and much effort will be needed to meet elimination targets.

**Figure 1.**
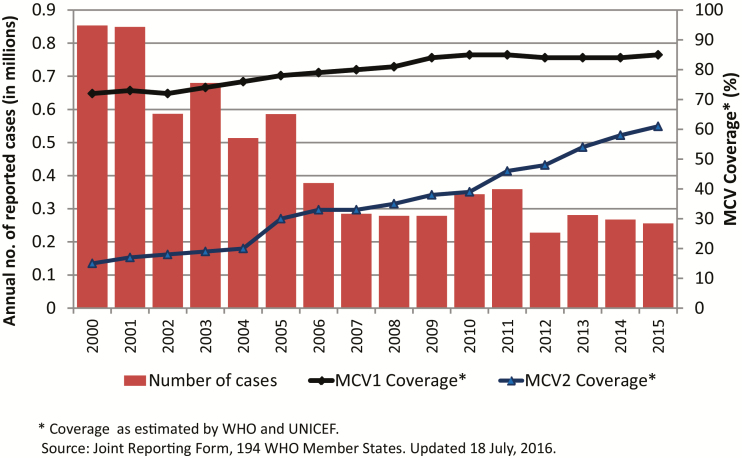
Annual reported measles cases and estimated coverage with the first dose of measles-containing vaccine (MCV1) and MCV2, 2000–2015. Coverage data were estimated by the World Health Organization (WHO) and the United Nations Children’s Fund (unpublished data, WHO Joint Reporting Form, 18 July 2016). These data elements to create this figure are available at the WHO IVB data site: http://www.who.int/immunization/monitoring_surveillance/data/en/. Acccessed 19 April 2017.

**Figure 2.**
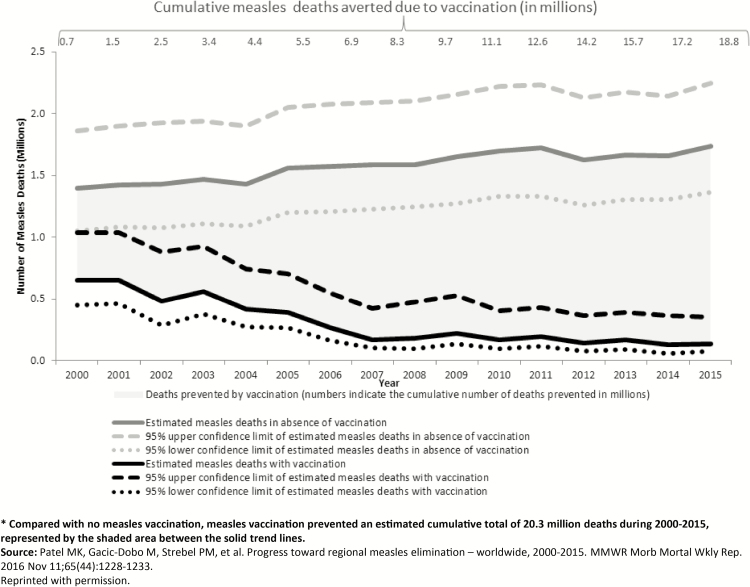
Global estimated number of measles deaths in the presence and absence of vaccination, 2000–2015. Compared with no measles vaccination, measles vaccination prevented an estimated cumulative total of 20.3 million deaths during 2000–2015, represented by the shaded area between the solid trend lines. Adapted with permission from the article by Patel et al [4].

During 2000–2015, the number of measles cases reported annually decreased worldwide from 853 479 to 254 928 (Figure 1), and annual reported measles incidence declined 75% worldwide, from 146 to 36 cases per million population [4]. Annual estimated measles deaths declined 79%, from 651 600 to 134 200 (Figure 2), and the decrease in measles mortality was among the main contributors to the decline in overall child mortality and progress toward MDG4 [10], with an estimated 20.3 million deaths averted during 2000–2015 [4]. Routine measles vaccination coverage, as measured by global coverage estimates from MCV1, increased globally during 2000–2015, from 72% to 85%, although coverage has remained at 84%–85% since 2009 (Figure 1). It should be noted that global coverage of routine MCV2 increased from 15% to 61% during 2000–2015, corresponding to an increase from 98 to 160 countries that introduced MCV2 during this period [4]. Estimated rubella-containing vaccine (RCV) coverage globally was 46% in 2015, with 147 countries having introduced RCV into their national immunization schedules [11]. While the European Region [12] and Western Pacific Region [13] have documented measles elimination in 21 and 5 countries, respectively, only 1 region, the Region of the Americas, has successfully achieved regional measles [14] and rubella [15] elimination.

Thus, while significant progress has been made toward achieving measles control and elimination targets, as well as other global child health milestones, much work remains to be done. As noted in a recent midterm review of the Measles and Rubella Global Strategic Plan 2012–2020 (commissioned by the Measles and Rubella Initiative Management Team, conducted by independent experts, and completed in 2016), “In principle, the 2020 goals can still be reached, but doing so would require a substantial escalation of political will and resources as well as heavy reliance on supplementary immunization activities [SIAs]” [16]. The authors of the review also note that the basic strategies articulated in the strategic plan are sound but that full implementation of the strategies is needed and has been limited by a lack of country ownership and global political will, as reflected in insufficient resources. As noted by the International Task Force on Disease Eradication (ITFDE), “Efforts to control and eliminate measles and rubella have accelerated incrementally since 2000, but have been greatly overshadowed in magnitude of resources and political commitment by GPEI. The impending completion of polio eradication opens a window of opportunity to devote greater attention to measles and rubella eradication” [10].

The feasibility and benefits of measles and rubella elimination have been well documented. In 2015, the ITFDE reinforced the advantages of pursuing measles and rubella eradication simultaneously and the need to make resources available for this effort [10]. Measles remains a major cause of childhood mortality [4], and rubella remains the leading infectious cause of birth defects, with >100 000 infants born with congenital rubella syndrome every year, mostly in low-income countries that have not yet introduced RCV [5]. The continuing morbidity and mortality burden of both diseases is unacceptable, owing to the availability of highly effective and inexpensive vaccines. The Strategic Advisory Group of Experts on Immunization has recommended that routine MCV2 should be added to all national immunization schedules [17]. The effectiveness of MCV1 is 93%–95% when administered to children ≥12 months of age and 85%–90% when administered to infants 9 months of age, and the effectiveness 97% for children ≥12 months of age at the time of the second doses of MCV given at least 28 days apart. Although antibody levels following vaccination may decline over time, measles and rubella vaccine–induced immunity provides long-term and likely lifelong protection. Measles is highly contagious, with a basic reproduction number (R_0_) of 12–18. Thus, 2 doses of measles vaccine are needed to reach the 89%–94% population immunity threshold required to prevent sustained measles virus transmission [18]. The effectiveness of a single dose of RCV is approximately 95% among infants 9 months of age. Since RCV is coadministered with MCV as a combined vaccine, the same strategies for vaccine delivery apply to both diseases. With the introduction of RCV in an increasing number of countries globally, strategies for measles elimination have the additional benefit of simultaneously advancing measles and rubella elimination goals. Rubella is less contagious than measles, with an R_0_ of 6–7; therefore, the biggest challenge to measles and rubella elimination efforts is the contagiousness of measles virus. However, both diseases are preventable with 1–2 doses of vaccine, have clinical symptoms that are easily detected through case-based surveillance, have no known animal reservoirs, and have relatively short incubation and transmissibility windows (Table 1).

**Table 1.** Parameters for Eradication of Vaccine-Preventable Diseases

**Parameter**	**Smallpox**	**Polio**	**Measles**	**Rubella**
Eradication status	Eradicated	Wild polio virus type 2 eradicated, type 3 potentially eradicated, and type 1 nearly eradicated	Candidate for eradication	Candidate for eradication
Clinical presentation	Fever and rash	Acute flaccid paralysis	Fever and rash	Fever and rash
Asymptomatic infections or carriers	No	Yes	No	No
Primary mode of transmission	Respiratory droplets	Fecal–oral route or oral–oral route	Aerosolized respiratory secretions	Aerosolized respiratory secretions
Period of contagiousness, d	25	28–42	9	1–5
Basic reproduction number	5–7	4–13	12–18	6–7
Herd or population immunity threshold, %	80−85	75−92	92–94	83–85
Serotypes	1	3	1	1
Vaccine delivery	Intradermal injection	Oral drops (oral polio vaccine) or intradermal or intramuscular injection (inactivated polio vaccine)	Subcutaneous injection	Subcutaneous injection
Vaccination strategy	Ring vaccination	Multiple repeated mass campaigns	Two doses of measles-containing vaccine through routine immunization, supplemented by periodic mass campaigns	One dose of rubella-containing vaccine through routine immunization, supplemented by periodic mass campaigns
Vaccine doses needed to stop transmission, no.	1	≥3	1–2	1
Vaccine-derived virus transmission	No	Yes	No	No

Global measles and rubella eradication will ultimately be cost saving, compared with other strategy scenarios of sustained control [8, 19]. High control, the policy currently pursued by global partners, costs governments and donors $2.3 billion per year for the foreseeable future, while still leading to >100 000 estimated measles deaths and >100 000 CRS cases annually [4,5]. Global measles and rubella eradication would save the current estimated treatment costs for measles virus and rubella virus infections ($8 billion per year) and prevent disability-adjusted life-year losses ($88 billion per year) [8].

The fundamental approach to measles and rubella elimination includes the following 5 strategies: achieving and maintaining high vaccination coverage with 2 doses of MCV, performing effective disease surveillance, developing and maintaining outbreak preparedness and response activities, communicating with and engaging stakeholders, and implementing research and innovations to improve the program [20]. The strategies used for polio eradication are similar and include performing high-quality real-time surveillance and creating and maintaining state-of-the-art global laboratory networks, preparing for and responding to outbreaks, performing periodic SIAs to reach inaccessible children, offering vaccine to every eligible child, monitoring programs and using accountability frameworks, using communication and social mobilization networks to generate demand for vaccine, and establishing and maintaining partnership coordination, advocacy, and resource mobilization activities. In addition, the framework and mechanisms of polio certification, at the country and regional levels, is providing a model for measles and rubella elimination verification processes. Currently, in many countries, polio and measles and rubella staff are the same people or interchangeable, working together in an interconnected fashion, often supported by existing polio assets. Work activities with considerable overlap in technical requirements and covered by the same staff include field and laboratory surveillance activities, case investigations, outbreak investigation and response, and SIA planning and implementation.

The global and regional needs for measles elimination overlap geographically with those of the 16 priority countries for polio transition, where GPEI assets are concentrated and the highest burden of measles cases and deaths, as well as rubella and CRS cases, occur [21]. The 16 countries with 95% of polio assets and infrastructure are Afghanistan, Angola, Bangladesh, Cameroon, Chad, the Democratic Republic of the Congo (DRC), Ethiopia, India, Indonesia, Myanmar, Nepal, Nigeria, Pakistan, Somalia, Sudan, and South Sudan. Among the estimated 20.8 million infants who did not receive MCV1 through routine immunization services in 2015, approximately 11 million (53%) were in the following 6 polio transition priority countries: India (3.2 million), Nigeria (3 million), Pakistan (2 million), Indonesia (1.5 million), Ethiopia (0.7 million), and the DRC (0.6 million) [6].

The Region of the Americas has already demonstrated how to successfully pivot from using resources for polio eradication and outbreak response to harnessing these assets for measles and rubella elimination. After wild poliovirus was declared eradicated in the Americas in 1994, the region maintained its investments, applying the polio lessons learned for similar strategies for the elimination of measles and rubella by achieving high population immunity and maintaining excellent surveillance [22]. The strategy for achieving high population immunity involved full implementation of a so-called catch-up, keep-up, follow-up strategy, in which SIAs targeting wide age ranges were followed by maintenance of high routine coverage and periodic follow-up SIAs for children aged 1–5 years [23]. In addition, the goal of measles and rubella elimination in the Americas benefitted from high-level political support. The last cases of endemic measles and endemic rubella occurred in 2002 and 2009, respectively, and the region is now verified as having eliminated measles and rubella [14,15]. The process of measles and rubella elimination provided opportunities for the countries in the region to strengthen health systems and thus ultimately to reduce health inequities [24]. The same experience can and should be replicated globally.

## MEASLES AND RUBELLA CONTROL AND ELIMINATION EFFORTS CURRENTLY DEPEND HEAVILY ON POLIO ASSETS

Many polio eradication assets and lessons learned have already been applied to measles and rubella elimination efforts. These VPD eradication and elimination efforts have similar strategies and program implementation infrastructure needs; therefore, polio eradication activities have easily been integrated with measles immunizations and surveillance activities [10]. Today, measles and rubella elimination efforts rely heavily on GPEI assets, including the staff, physical infrastructure, and financing that are used to support the program. A 2014 survey of 467 country-level program managers in 10 countries (Afghanistan, Angola, Chad, the DRC, Ethiopia, India, Nigeria, Pakistan, Somalia, and South Sudan) found that, overall, they spent 22% of their time on routine immunization and 46% of their time on immunization goals and activities beyond polio. They also reported spending 8% of their time on measles and rubella, highlighting the important support GPEI assets are currently providing to measles and rubella control and elimination activities [21]. The heavy dependence of EPI on GPEI resources was highlighted in pilot case studies in the DRC and Nepal. In the DRC, staff observed that, “without polio, the whole health system would suffer” [25]. GPEI staff provided technical and operational support for EPI, such as support for national planning, technical assistance and training of national EPI staff, and participation in RI management, including supply chain management for vaccine procurement and cold chain maintenance. The consequences of losing polio assets would include the likely reversal of EPI progress in the 16 priority countries, as well as globally. Currently, GPEI assets are budgeted to taper off through 2019. Transitioning assets and finding alternative sources of funding are critically needed to maintain the progress to date and accelerate the progress toward GVAP goals, including measles and rubella elimination.

Surveillance for VPDs, particularly measles and rubella, is heavily dependent on GPEI resources. Overall, GPEI funds approximately $140 million annually on all surveillance activities, for WHO personnel and operational costs, in polio-endemic and -nonendemic countries. GPEI surveillance officers, particularly in polio-nonendemic countries, play a critical role in measles and rubella fever/rash surveillance [26]. For example, in the DRC, GPEI-funded WHO surveillance teams spent approximately one quarter of their time on surveillance of other infectious diseases [21]. Staff noted that the GPEI provides tools, training, equipment, and funding for local surveillance in health districts and that technical assistance to polio laboratories benefitted national laboratories with best practices shared across disease areas. In Nepal, the polio-funded WHO Immunization Preventable Disease program is the backbone of disease surveillance activities and the primary surveillance program for VPDs.

Estimates of the global costs for fully financing measles and rubella surveillance would require replacing these officers, and economic estimates for fully financed global measles and rubella surveillance place the annual cost at $60 million [19]. Current funding dedicated to measles and rubella surveillance from global partners, including the Centers for Disease Control and Prevention, the Measles and Rubella Initiative, the Bill and Melinda Gates Foundation, and Gavi, the Vaccine Alliance, is approximately $35 million. Thus, a portion of the gap between current measles and rubella surveillance funding and the total cost of fully funding measles and rubella surveillance is provided through reliance on and integration with GPEI laboratory assets; this support is threatened with the scaling down of polio infrastructure.

GPEI and Measles and Rubella Initiative partner investments over decades have built up robust surveillance systems for acute flaccid paralysis, to detect polio, and for rash/fever-associated illness, to detect measles and rubella. These 2 systems are supported by the Global Polio Laboratory Network and the Global Measles and Rubella Laboratory Network (GMRLN), which was built onto the Global Polio Laboratory Network platform; considerable overlap exists with shared staff, management, and resources for these 2 surveillance systems, with support from the GPEI and the Measles and Rubella Initiative. GPEI assets include large networks of surveillance officers; processes for specimen collection, transport, and testing; as well as systems for data management, analysis, and use. The GMRLN surveillance system provides a platform for detecting other viral VPDs, such as yellow fever, as well as emerging diseases, such as Ebola. The global Ebola emergency response relied on existing eradication infrastructure and demonstrated the critical importance of maintaining and building upon this capacity by transitioning GPEI assets to elimination efforts involving laboratory-based surveillance, outbreak detection and response, and the ability to implement mass vaccination campaigns [27].

## FOCUSED EFFORTS ON MEASLES AND RUBELLA ELIMINATION CAN PROVIDE SYNERGY AND BENEFITS FOR BROAD IMMUNIZATION AND SURVEILLANCE ACTIVITIES

Applying polio assets to measles and rubella elimination will benefit immunization systems because, unlike polio eradication, measles elimination requires high 2-dose coverage through routine immunization service delivery, combined, in some settings, with periodic mass vaccination campaigns every 3–5 years. Because of the highly effective measles and rubella vaccine, repeated multiple rounds of mass vaccination targeting the same age groups will not be required, as was needed with the use of oral polio vaccine for polio eradication. Thus, measles elimination efforts can take advantage of vertical strategies that focus on using surveillance data for action and measles outcomes to identify areas missed by vaccination and horizontal strategies that build systems and health services to sustain the gains and achieve broader objectives. The combination of these approaches has been described as a “diagonal approach” [28].

Measles elimination efforts incorporating a comprehensive approach for achieving high 2-dose MCV coverage improves EPI coverage broadly and strengthens the immunization system. Implementation of this strategy provides a platform for routine and mass delivery of other immunizations and child-survival interventions. The focused approach that is needed in eradication efforts has provided methods of better using surveillance data and coverage data to identify areas of low vaccination coverage, instilling a data-driven approach to immunization and surveillance that increases immunization coverage and equity in the entire population, including those living in hard-to-reach places. The identification of susceptible populations and the emphasis on mapping and reaching all communities with vaccine, which is necessary for eliminating chains of transmission from all reservoirs, has proven valuable for strengthening immunization service delivery and other public health activities. The experience in the United States has demonstrated the value of measles elimination efforts in strengthening the entire immunization system [29]. The value of focused eradication/elimination efforts should not be underestimated, and further investments in these efforts will lead to achieving high coverage and equity for all immunizations and other public health initiatives. The inextricable linkage between achieving and maintaining measles and rubella elimination and strong immunization systems is repeatedly underlined in the final report of the Midterm Review of the Global Measles and Rubella Strategic Plan [16].

The far-reaching approach of focused eradication/elimination programs extends to surveillance. Disease eradication efforts require high-quality surveillance covering entire populations. The goal-driven measles and rubella elimination activities, including detailed outbreak investigations, often provide important information about program failures that lead to solutions for achieving high immunization coverage and equity. Because measles vaccine is highly effective and measles virus is highly contagious, measles is used as an indicator of program performance and can serve as a proxy for identifying weaknesses of an immunization program. Measles can serve as an indicator of the strength and reach of the health system [29]. When there are gaps in immunization coverage, measles is most often the first VPD one sees, indicating low immunization coverage. Analysis of measles surveillance data identifies populations and areas where immunization coverage is suboptimal and thereby helps guide efforts to strengthen immunization service delivery.

## TRANSITIONING POLIO RESOURCES TO MEASLES AND RUBELLA ELIMINATION EFFORTS WILL HELP ENSURE THE ONGOING SUCCESS OF POLIO ERADICATION EFFORTS

The GPEI built an infrastructure for eradicating polio that can now be retooled for elimination efforts for other VPDs. GPEI assets are well positioned to support efforts to achieve the measles and rubella elimination goals that have been set by the countries of each region of the world while maintaining the essential polio functions, such as polio surveillance, that will continue to be needed after the world is certified to be free of polio. The Midterm Review of the Global Measles and Rubella Strategic Plan noted that, at a minimum, there should be no weakening of nonpolio activities currently supported by polio assets [16].

During the first few years following the last reported polio case, in addition to polio containment activities, there will be a need to maintain high routine immunization coverage, surveillance, and capacity for outbreak response, communications, and mass vaccination campaigns following the conclusion of the GPEI. The current measles and rubella elimination infrastructure is a natural fit to support these activities. Maintaining and mainstreaming essential polio eradication functions, such as immunization, surveillance, communication, response, and containment, into ongoing public health programs will be critical for continuity of services in a polio-free world following the conclusion of the GPEI. These functions will still be required after polio eradication is certified globally. Countries and partner organizations must ensure that these functions continue and are mainstreamed into appropriate ongoing public health programs that have components that are aligned with these needs. Eradication of measles and rubella would require a sustained global commitment and a clear accountability framework similar to those that exists in the GPEI.

## CONCLUSION

As the GPEI scales down, it is imperative that we collectively use our forward vision to capitalize on the experience, momentum, and infrastructure developed over nearly 30 years to eradicate polio. A transition to measles and rubella elimination, with a mutually reinforcing synergy with systems strengthening and routine immunization, is the ideal next phase.

It will be critical to incorporate the many lessons learned from polio eradication. These include the successes, failures, innovations, and best practices that the polio program has identified over the last 3 decades that enable the assets and functions to provide more-efficient, equitable, high-quality services, particularly to underserved, insecure, hard-to-reach, or high-risk communities. In many areas, the primary challenges were the poor quality of the program, the failure to reach every child, and the repeated missing of underserved populations. In particular, the program identified innovative strategies to work in insecure and inaccessible areas. On the management side, 5-year plans and funded budgets have been critical for programs to manage their resources effectively; chronic funding gaps meant that vulnerable countries were unable to conduct planned SIAs, which led to outbreaks and delays in eradication and insufficient population immunity to prevent circulation of reintroduced virus. One of the most important lessons from the experience with polio is the value of prevention. Outbreaks are far more expensive than prevention activities. Thus, stopping endemic transmission once was not enough in some countries, demonstrating that it was essential to achieve and maintain high population immunity.

Until polio eradication is achieved, the world might not be ready to commit to another global eradication goal. However, starting the transition of assets to existing elimination efforts will help further progress toward achieving the established regional goals for measles and rubella elimination. GPEI assets should go to support full implementation of the recommended measles and rubella elimination strategies to identify and interrupt measles and rubella virus transmission in major reservoirs, further reduce measles mortality, improve immunization service delivery to achieve high coverage and equity, and maintain an eradication/elimination infrastructure for conducting mass vaccination campaigns and global laboratory-supported surveillance systems. Ridding the world of polio will reduce the burden on public health programs; there will no longer be a need to dedicate resources or manpower to controlling and responding to smallpox or polio. Of course, the real impact and legacy of disease eradication is the impact that it has on improving the lives of everyone. Eradication provides true health equity for all and forever. With polio assets, we can implement the recommended strategies for measles and rubella elimination, achieve equitable access to vaccination services, and achieve GVAP and regional elimination goals and the eventual goal of global measles and rubella eradication.

## Notes

***Supplement sponsorship.*** This work is part of a supplement coordinated by the Task Force for Global Health with funding provided by The Bill and Melinda Gates Foundation and the Centers for Disease Control and Prevention.

***Potential conflicts of interest.*** All authors: No reported conflicts of interest. All authors have submitted the ICMJE Form for Disclosure of Potential Conflicts of Interest. Conflicts that the editors consider relevant to the content of the manuscript have been disclosed.
